# Liquid Chromatography–Tandem Mass Spectrometry in Newborn Screening Laboratories

**DOI:** 10.3390/ijns8040062

**Published:** 2022-11-28

**Authors:** Michael H. Gelb, Khaja Basheeruddin, Alberto Burlina, Hsiao-Jan Chen, Yin-Hsiu Chien, George Dizikes, Christine Dorley, Roberto Giugliani, Amy Hietala, Xinying Hong, Shu-Min Kao, Hamid Khaledi, Tracy Klug, Francyne Kubaski, Hsuan-Chieh Liao, Monica Martin, Adrienne Manning, Joseph Orsini, Yin Peng, Enzo Ranieri, Andreas Rohrwasser, Nicolas Szabo-Fresnais, Coleman T. Turgeon, Frédérick M. Vaz, Li-yun Wang, Dietrich Matern

**Affiliations:** 1 Departments of Chemistry and Biochemistry, University of Washington, Seattle, WA 98195, USA; 2 Newborn Screening Laboratory, Illinois Department of Public Health, Chicago, IL 60612, USA; 3 Division of Inherited Metabolic Diseases, Reference Centre Expanded Newborn Screening, University Hospital Padova, 35128 Padova, Italy; 4 The Chinese Foundation of Health, Neonatal Screening Center, Taipei 110, Taiwan; 5 Department of Medical Genetics and Pediatrics, National Taiwan University Hospital and National Taiwan University College of Medicine, Taipei 100, Taiwan; 6 Tennessee Department of Health, Division of Laboratory Services, Knoxville, TN 37920, USA; 7 Department of Genetics UFRGS, Medical Genetics Service, HCPA, DASA and Casa dos Raros, Porto Alegre 91501970, Brazil; 8 Newborn Screening Laboratory, Minnesota Department of Health, St. Paul, MN 55164, USA; 9 GelbChem LLC, 4000 Mason Road, #300, Seattle, WA 98105, USA; 10 Missouri Department of Health and Senior Services, P.O. Box 570, Jefferson City, MO 65102, USA; 11 Department of Laboratory Medicine and Pathology, University of Washington, Seattle, WA 98195, USA; 12 Wadsworth Center, New York State Department of Health, Newborn Screening Program, David Axelrod Institute, 120 New Scotland Ave., Albany, NY 12201, USA; 13 Connecticut Department of Public Health, Katherine A. Kelley Public Health Laboratory, Rocky Hill, CT 06067, USA; 14 Genetics and Molecular Pathology, SA Pathology (WCH Site), Adelaide 5043, Australia; 15 Utah Department of Health and Human Services, Salt Lake City, UT 84129, USA; 16 Department of Laboratory Medicine and Pathology, Biochemical Genetics Laboratory, Mayo Clinic, Rochester, MN 55902, USA; 17 Laboratory Genetic Metabolic Diseases, Academic Medical Center, 1105 AZ Amsterdam, The Netherlands; 18 Taipei Institute of Pathology, Taipei 103, Taiwan

**Keywords:** newborn screening, tandem mass spectrometry, liquid chromatography, dried blood spots, inborn errors of metabolism, reflex testing

## Abstract

Tandem mass spectrometry (MS/MS) is the most universal platform currently available for the analysis of enzymatic activities and biomarkers in dried blood spots (DBS) for applications in newborn screening (NBS). Among the MS/MS applications in NBS, the most common is flow-injection analysis (FIA-) MS/MS, where the sample is introduced as a bolus injection into the mass spectrometer without the prior fractionation of analytes. Liquid chromatography combined with MS/MS (LC-MS/MS) has been employed for second-tier tests to reduce the false-positive rate associated with several nonspecific screening markers, beginning two decades ago. More recently, LC-MS/MS has been applied to primary screening for new conditions for which FIA-MS/MS or other methods, including genomic screening, are not yet adequate. In addition to providing a list of the currently used LC-MS/MS-based assays for NBS, the authors share their experience regarding the maintenance requirements of LC-MS/MS vs. FIA-MS/MS systems. The consensus is that the maintenance of LC-MS/MS and FIA-MS/MS instrumentation is similar, and LC-MS/MS has the advantage of allowing for a larger number of diseases to be screened for in a multiplex, cost-effective fashion with a high throughput and an adequate turnaround time.

## 1. Introduction

The introduction of triple quadrupole tandem mass spectrometers in clinical laboratories revolutionized the ability to carry out newborn screening (NBS) for multiple diseases in a single multiplexed analysis using dried blood spots (DBS) [1]. For NBS, tandem mass spectrometry (MS/MS) applications have so far relied on electrospray ionization to introduce gas phase analytes from extracted DBS into the MS/MS instrument. Once introduced, the first quadrupole (Q1) is set up to either detect the ions’ mass over a predetermined mass range or for multiple ions based on their predicted mass. Following ion fragmentation in Q2, Q3 is set to measure one or more analyte-specific fragment ion mass. This allows for the identification and measurement of the abundance of specific ions derived from the intact analyte of interest. For most biomarkers, precursor and fragment ion pairs are sufficiently specific for the targeted analyte(s), providing rapid and interference-free analysis of multiple markers in a single analysis.

In the early employment of MS/MS to NBS, the extract of a 3 mm punch from a DBS is infused into the mass spectrometer in the absence of the chromatographic separation of analytes. This is called flow-injection analysis MS/MS (FIA-MS/MS). To the DBS extract, internal standards are added at known concentrations. These are chemically identical to the analytes of interest but are labeled with heavy isotopes, allowing for the quantification of analytes by the comparison of the ratio of the MS/MS abundance signal for the analyte to that of the internal standard. The use of internal standards allows the MS/MS detector response to be converted to moles of analytes. Each analyte and its companion internal standard are detected by a precursor ion scan, neutral loss scan and/or multiple reaction monitoring (MRM) that involves mass filtering for the parent ions (in Q1) followed by the collision of these ions with inert gas to form fragment ions (in Q2), which are mass-selected prior to detection in Q3. In the MRM mode, the mass spectrometer cycles through a list of MRMs such that each analyte and internal standard are separately detected in rapid succession.

More recently, FIA-MS/MS multiplex methods have been developed for the NBS of lysosomal diseases (LSDs) [2]. These assays measure the enzymatic products produced after the incubation of a collection of enzyme substrates with a punch from the DBS, allowing for the calculation of enzyme activity in the DBS. This contrasts with the first MS/MS applications in NBS, which measure the endogenous metabolites present in the DBS. As is true for any enzyme assay, they rely on carefully designed buffers that contain one or more enzyme specific substrate and provide an environment that allows for the formation of enzyme-specific product(s) that can be detected and quantified accurately. In the current MS/MS-based lysosomal enzyme assays, a DBS punch is incubated, typically overnight, with a buffer that does not require the extraction of an enzyme prior to incubation. The enzyme product(s) can be quantified by FIA-MS/MS using isotopically labeled internal standards which are also included in the incubation buffer.

The FIA-MS/MS method has been modified in some laboratories by passing the sample mixture through a high-pressure liquid chromatography (LC) column prior to MS/MS (LC-MS/MS) [3,4]. FIA-MS/MS is sufficiently sensitive to detect products of enzyme assays in screening for several LSDs—for example, mucopolysaccharidoses type I (MPS-I) and Pompe disease, which have been included as core conditions in the Recommended Uniform Screening Panel (RUSP) in the USA. MPS-II was recently added to the RUSP and currently requires LC-MS/MS to measure the activity of iduronate-2-sulfatase when MS/MS is the preferred analytical platform vs. fluorometric methods (see below).

NBS programs currently use MS/MS for the detection of markers of more disorders than any other technique (e.g., fluorometric assays, PCR-based assays, immunoassays and electrophoretic assays). MS/MS provides the most general biochemical method for the detection of analytes in DBS and allows for a high degree of multiplexing such that many analytes can be extracted from one DBS punch and quantified in a single infusion into the mass spectrometer. Increasing MS/MS assays for NBS will require that FIA-MS/MS continues to be expanded and that LC-MS/MS is added to the NBS laboratory workflow. Generally, it is not very complicated to add another MS/MS detection channel to an existing assay so that a new analyte/internal standard pair can be measured. In addition, biochemical assays requiring separate pre-MS/MS processing of multiple DBS punches can be combined for a single infusion for either LC-MS/MS or FIA-MS/MS analysis.

In this review, we focus on the likely expansion of the use of MS/MS by NBS programs by the incorporation of LC-MS/MS in addition to FIA-MS/MS. This has already occurred in NBS laboratories represented by the authors of this article. We also provide information on LC-MS/MS equipment maintenance for NBS and preview why the further expansion of NBS is likely to include additional LC-MS/MS assays. Of course, the expansion of NBS is not, and should not be, driven by the development and availability of new analytical techniques. However, since Dr. Robert Guthrie started NBS for phenylketonuria 60 years ago, it has been true that improved and new assay technologies are a prerequisite before conditions for which interventions exist that benefit the newborn and their families can be added to NBS programs.

## 2. Comparison of FIA-MS/MS and LC-MS/MS

LC-MS/MS has some advantages over FIA-MS/MS (Table 1): (1) The pre-MS/MS sample cleanup steps are often no longer needed when LC-MS/MS is used, since most of the unwanted material elutes in the void volume of the LC column and can be diverted by a flow line valve to waste rather than to the mass spectrometer. This lessens the need to clean the electrospray ionization source of the mass spectrometer, as was shown in FIA-MS/MS versus LC-MS/MS comparison studies [5]. (2) Analytes are concentrated into small volumes by LC, which allows for the detection of low-abundance analytes that would not otherwise be detected by FIA-MS/MS [6,7]. (3) The use of LC rather than FIA avoids the problem of the in-source breakdown of enzymatic substrates to enzymatic products in the electrospray source. Heat-labile substrates—for example, sulfatase substrates that contain a sulfate ester-undergo a partial loss of the sulfate in the electrospray source, thus giving rise to enzyme-independent product formation (higher background). Since enzyme substrates and products are separated by LC prior to MS/MS, in-source breakdown is not an issue, since only the material eluting at the product retention time is quantified (along with the internal standard). (4) Often, there are isobaric interferences such that the MRM conditions are not specific for the analyte of interest. LC usually leads to a resolution of the analyte from the interfering agents prior to MS/MS. 

The stated disadvantages of LC-MS/MS versus FIA-MS/MS (Table 1) are: (1) LC-LC-MS/MS is more time-consuming than FIA-MS/MS. (2) LC-MS/MS is more complex than FIA-MS/MS in that additional equipment is required, and, with it, additional equipment maintenance is required. However, the additional equipment required is minimal, and concerns about increased maintenance have not materialized, as documented by several laboratories represented by the authors of this review (see below). (3) The software-based integration of analyte peaks is more complicated than that with FIA-MS/MS because the peak boundaries must be chosen properly. This is not always achieved in every run using automated integration, and, thus, some user-inspection of the integration may be required. Further optimization of automated LC-MS/MS peak integration would be warranted. Additional technological advances have decreased the analytical time per sample for LC-MS/MS, which is now comparable to that of FIA-MS/MS.

## 3. LC-MS/MS Assays Currently in Use for Routine NBS

Several LC-MS/MS assays are now employed by NBS programs for second-tier testing (Table 2). These were initially developed to reduce the number of false-positives for conditions where the primary screening marker’s reference range and disease range overlap [8]. The first such LC-MS/MS assay employed for NBS was a steroid profile used as a second-tier test to reduce the false positive rate of NBS for congenital adrenal hyperplasia [9,10]. The concept of taking advantage of the higher resolution and sensitivity afforded by LC-MS/MS over FIA-MS/MS and the ability of MS/MS to simultaneously measure multiple biomarkers led to the development and implementation of several more second-tier tests to improve the specificity of NBS for other conditions affected by low positive predictive values, such as inborn errors of amino acid, fatty acid and organic acid metabolism (Table 2).

LC-MS/MS analysis of these markers may not be appropriate for first-tier NBS because of the long analytical times (several minutes per sample) and, often, the need for top-end MS/MS instrumentation (in terms of signal-to-noise) that must be maintained in ultra-clean conditions. While molecular genetic second-tier testing is also used by some NBS programs, the biochemical assays are currently generally superior in terms of turnaround, cost and phenotype prediction. These tests are performed on the original DBS, and a normal result of the second-tier test overrules the first-tier test result. They can be performed by each NBS laboratory or sent to another lab. However, timeliness must be considered because some disorders are time-critical, making rapid turnaround and overnight shipping necessary. Table 2 lists LC-MS/MS assays that are now used as part of NBS and whose utility in improving the performance of the NBS program has been documented.

For the NBS of MPS-I by first-tier enzymatic activity assays, a large proportion of below-cutoff enzyme activities are samples due to pseudodeficiencies, which, if reported would be shown by diagnostic testing to be false-positives. The measurement of glycosaminoglycans in a separate punch from the same DBS readily separates true deficiencies from pseudodeficiencies [11,12]. The same is true for the NBS of MPS-II [13]. Likewise, the measurement of psychosine in DBS greatly reduces false-positives for Krabbe disease, which made it more likely that the nomination of Krabbe disease for inclusion on the RUSP would be successful [14,15]. While currently not applicable to first-tier, population NBS, it has been shown, particularly for Krabbe disease, that these assays are superior to second-tier DNA analysis in terms of phenotype prediction and should be included as part of NBS to minimize the unnecessary follow-up for families that is always accompanied by anxiety and costs [16]. Using the second-tier LC-MS/MS assays, false-positives for LSDs are among the lowest of those observed in all other NBS conditions currently screened for [17].

The first assay to use LC-MS/MS to quantitate enzyme products brought into use in an NBS laboratory as a primary screening test is the 6-plex LSD assay (Pompe, MPS-I, Krabbe, Fabry, Niemann-Pick-A/B, and Gaucher) used by the Illinois NBS laboratory, which was recently expanded to assay seven lysosomal enzymes with the addition of MPS-II [19,20,32]. One 3 mm DBS punch is incubated in an assay cocktail for all enzymes except iduronate-2-sulfatase for MPS-II. A second 3 mm DBS punch is used for the MPS-II assay. After the enzymatic reactions are stopped, the two assay mixtures are combined and analyzed in a single LC-MS/MS run per newborn, with an inject-to-inject time of 2.1 min.

NBS in Taiwan is carried out by three regional laboratories, and all use LC-MS/MS to screen for MPS-I, MPS-II, MPS-IVA and MPS-VI; two of the three NBS labs also include MPS-IIIB and CLN2 (Table 2). All screen for Pompe, Fabry and Gaucher (2/3 by FIA-MS/MS and one out of three includes these in the LC-MS/MS run with MPSs [33]).

In the US, MPS-II was added to the RUSP in August 2022. Illinois and all three NBS laboratories in Taiwan screen for MPS-II in their multiplexed LC-MS/MS assays (Table 2). FIA-MS/MS has been shown to not be appropriate for the measurement of iduronate-2-sulfatase activity because its substrate undergoes significant breakdown to its product by in-source fragmentation alone. This is of no concern when LC-MS/MS is used because the substrate and enzymatic product are well separated by liquid chromatography. As an alternative approach, the state of Missouri has been using fluorometry measurement in a standalone enzyme assay to screen for MPS II since 2018 [34].

NBS for X-ALD is carried out by the measurement of lysophosphatidylcholines (LPC), particularly C26-LPC. This is possible by FIA-MS/MS in combination with the analysis of amino acids, acylcarnitines and succinylacetone [35] or by combining the LPC analysis with lysosomal enzyme assays [22]. With C26-LPC measured first with FIA-MS/MS, above-cutoff results need to be re-analyzed by a second-tier LC-MS/MS assay because the FIA-MS/MS method is not analytically specific for the C26-LPC analyte. Some NBS labs have opted for the standalone first-tier LC-MS/MS analysis of C26-LPC only (Table 2), a questionable approach given the lack of multiplexing and thus the associated increase in cost.

Utah is in the process of implementing a 3-plex method using LC-MS/MS for the NBS of X-ALD, Pompe disease and MPS-I, with an instrument time of ~2 min per sample. As previously published, this has the advantage of avoiding a separate set of MS/MS instruments—one being an FIA-MS/MS system for Pompe disease and MPS-I, and the other being an LC-MS/MS system for X-ALD [36].

Archimedlife is a private company in Austria that provides NBS by contracts with several large hospitals in Europe (https://www.archimedlife.com/, accessed on 10 June 2022). The conditions comprise an expansion of NBS beyond government-mandated panels. This includes the first prospective, routine NBS program for metachromatic leukodystrophy (MLD) along with several other LSDs. Many of the employed assays are performed by first-tier multiplexed LC-MS/MS-based methods. 

## 4. Conditions Particularly Amenable to LC-MS/MS Analysis for NBS

There are a number of conditions that are now being considered for NBS, for which LC-MS/MS appears to be the most reasonable analytical approach. One example is MLD due to the deficiency of arylsulfatase A, which removes sulfate from the sphingolipid sulfatide. Arylsulfatase A was shown to be insufficiently stable in DBS, rendering the measurement of enzymatic activity problematic as a first-tier NBS method [6,37]. One solution is to measure the accumulation of the sulfatide substrate in DBS, followed by the measurement of the sulfatase activity or the molecular genetic analysis of *ARSA* as a second-tier test(s) [6]. Sulfatides cannot be measured by FIA-MS/MS because of their low abundance, but they can readily be detected by LC-MS/MS [6]. The addition of MLD to NBS programs has gained increased interest because of the successful treatment afforded by gene therapy combined with hematopoietic stem cell transplantation [38].

Another example where only MS/MS currently provides an NBS solution is cerebrotendinous xanthomatosis (CTX), a treatable disease due to the deficiency of a cytochrome P450 enzyme in the bile acid biosynthetic pathway. One reported biochemical assay employs MS/MS to measure a bile alcohol, cholestanetetrol glucuronide, in DBS [39]. A research study of ~30,000 random newborn DBS for MLD and CTX was carried out using a multiplexed LC-MS/MS assay [40]. For each disease, a single-screen positive case was found, and the molecular genetic analysis of the relevant gene showed two pathogenic variants in both cases, suggesting a highly specific screening test [6,40]. Of note, a FIA-MS/MS assay may be possible for CTX, and a study is underway in The Netherlands which compares the LC-MS/MS and FIA-MS/MS methods (F. Vaz, Amsterdam UMC).

For Niemann-Pick C (NP-C), specific therapy with miglustat is available, and clinical trials of new therapies are underway. This LSD is due to the deficiency of a lysosomal lipid transporter, and the only published NBS option is the measurement of lipid biomarkers in DBS—most notably, bile acid B by LC-MS/MS [7]. Attempts to detect this analyte by FIA-MS/MS in the Gelb laboratory have been unsuccessful due to the low abundance of the biomarker in DBS.

An LC-MS/MS assay for measuring the activity of the enzyme relevant to MPS-IIIA was recently reported [41]. The activity of this enzyme is relatively low in DBS, and LC-MS/MS is required to provide added sensitivity over the FIA-MS/MS approach [41]. An immunocapture method has been reported [42], but no data for its use in NBS are available. In addition, a recent report states that several attempts using a fluorimetric approach have failed [43].

A recent application of LC-MS/MS to assays relevant to NBS is the use of the so-called Immuno-SRM method. Proteins in a DBS are first digested with trypsin, and the target peptide from the protein of interest is captured by a monoclonal antibody using magnetic bead technology. The peptide is released from the antibody and quantified by LC-MS/MS together with an isotopic-substituted peptide internal standard [44,45]. This has been applied to Wilson disease, which is due to a defective intra-cellular copper transporter and for which effective medical treatment has been available for decades [45]. Ceruloplasmin analysis by an immunoassay was suggested for the NBS of Wilson disease but was ultimately proved to be insufficiently specific [46]. LC-MS/MS, proteomic-type assays have also been developed for the detection of primary immunodeficiencies using DBS, where the disease is caused by a deficiency of a key protein required for immune development [44]. These types of proteomic assays may become useful in cases where a conventional immunoassay does not work using DBS samples.

## 5. Pilot Studies of LC-MS/MS-Based Assays for NBS

Multiplexed LC-MS/MS research studies have been carried out at the University of Washington using de-identified newborn DBS from the state’s NBS program. They first evaluated a single LC-MS/MS-based multi-enzyme assay on ~100,000 DBS for MPS-II, MPS-IIIB, MPS-IVA, MPS-VI and MPS-VII using a single DBS punch [5]. Later, a study was conducted using a 2-plex assay on ~30,000 DBS for MLD and CTX using a second DBS punch [6,40]. It is a relatively straightforward matter to combine these two methods into a single LC-MS/MS multiplex assay based on the reported 18-plex assay that includes all of these diseases [36]. As mentioned above, FIA-MS/MS and LC-MS/MS are currently being compared in a pilot study for CTX in the Netherlands. 

ScreenPlus is a prospective NBS study of consented neonates born in several hospitals in New York state [47]. Two LC-MS/MS multiplex assays (~2 min inject-to-inject for each assay) are performed in parallel in the New York NBS laboratory. One uses a single DBS punch to measure the activities of the enzymes deficient in MPS-II, MPS-IIIB, MPS-IVA, MPS-VI, MPS-VII, CLN2, lysosomal acid lipase, Gaucher, Fabry and Niemann-Pick-A/B. The second LC-MS/MS assay uses a second DBS punch which is extracted with methanol for biomarker analysis (NP-C, CTX and MLD) [36]. This is currently the largest consented study in the world in terms of the number of conditions being tested simultaneously. These LC-MS/MS assays are expected to be expandable to include other conditions for which the biomarkers can be added simply by turning on additional MRM channels.

One state in Brazil has performed research studies for six LSDs using FIA-MS/MS for Fabry, Gaucher, Pompe, MPS I, ASMD and Krabbe and is planning to expand to a 16-disease panel using LC-MS/MS (personal communications with R. Giugliani and F. Kubaski).

A highly multiplexed immunocapture method was proposed for the NBS of LSDs by Hopwood, but limited comparison studies have not shown an advantage of this approach over FIA-MS/MS or fluorometry-based methods [48]. Moreover, the necessary antibodies for this assay are not commercially available.

## 6. LC-MS/MS Equipment Maintenance

FIA-MS/MS and LC-MS/MS both require liquid streaming of the prepared sample into the electrospray source of the mass spectrometer. FIA-MS/MS typically is carried out with a single solvent composition delivered via a single medium pressure pump, whereas LC-MS/MS is typically carried out with a binary solvent gradient requiring two high-pressure pumps. Both make use of an essentially identical sample handler for the automated delivery of samples in a sequential fashion (Table 1). Modern LC pumps are very reliable, even with the higher pressures required for fast flow rates (~0.5 mL/min) of solvents through columns containing <2 micron stationary phase particles typically used for rapid and high-resolution chromatography.

A key advantage of LC-MS/MS over FIA-MS/MS is that a large fraction of contaminants present in DBS elute in the void volume of the LC column (no retention to the solid phase matrix) and are shunted away from the electrospray ionization source of the mass spectrometer with a diversion valve. This is not possible with FIA-MS/MS, since a single sample composition is infused over the entire analysis period. This valve shunting allows for less frequently needed cleaning of the electrospray source when using LC-MS/MS vs. FIA-MS/MS [5].

To obtain current information on the instrument maintenance of the LC-MS/MS platform, all authors provided instrument upkeep information for the LC-MS/MS assays employed in their NBS laboratory for at least 6 months (Table 3). Of note, none of the laboratories report issues that are specific to the LC component of the LC-MS/MS, indicating that LC-MS/MS and FIA-MS/MS have similar equipment maintenance requirements.

## 7. Multiplex LC-MS/MS Assays and/or Genomic Analysis for NBS

There is widespread discussion about an increasing use of next-generation sequencing (NGS) of DNA to support NBS and even to be used as the primary screening assay [49]. It is well established that NGS, for first-tier NBS, will lead to a large number of cases with genotypes containing variants of uncertain significance (VUS), as well as variants that lead to a partial reduction in the function of the encoded protein. Some of the latter may not cause disease alone but could contribute to disease when combined with other variants. While some DNA variants are predicted to cause a complete loss of function of the encoded protein, the vast majority are missense mutations for which the impact of the single amino acid substitution on protein function is difficult to predict.

The application of gene sequencing as a second-tier test to improve the NBS for Krabbe disease and other LSDs has already demonstrated limited value given the large number of VUS [50]. Indeed, of 996 reported variants in *GALC*, the gene encoding galactocerebrosidase that is deficient in Krabbe disease, only 300 variants (30%) are currently of certain significance [51]. Biochemical second-tier tests could limit false-positive results from NGS-based NBS; however, the extended analytical time for NGS alone would have to be improved for the NBS of such conditions that are extremely time-sensitive, i.e., where treatment initiation or planning needs to start within the first two weeks of life. For these reasons, it is likely that LC-MS/MS will be the analytical platform of choice to expand NBS efficiently and effectively for the foreseeable future. Should the NBS of a few or many conditions be carried out by first-tier NGS, LC-MS/MS biochemical assays will be needed to sort through first-tier positives with genotypes of uncertain significance.

## 8. Discussion

With the continued expansion of NBS panels, highly multiplexable, single analytical platform screening assays are preferred in order to minimize the hands-on effort by laboratory personnel and equipment needs and because the DBS sample is of limited quantity. Some of the multiplexed assays mentioned in this review are “multiplexed” in terms of providing a more efficient screening approach by including several biomarkers for multiple conditions. However, some of these assays fall short of the goal of needing only one 3 mm DBS punch but require two or more punches to extract and prepare specific markers for combined MS/MS analysis. This is true for both FIA-MS/MS and LC-MS/MS methods [20,22,36].

Large scale DNA sequencing technology is revolutionizing the diagnostic process for patients with inherited disorders of all ages. Combined with the constantly improving bioinformatic analysis of genomic data, newborns in an increasing number of neonatal intensive care units benefit from rapid exome and even genome analyses [52]. These advancements have led to the funding of research projects to evaluate genomic NBS. The results from a recent study led the authors to propose adding genomic analysis to complement but not replace MS/MS for NBS, but they also acknowledge that the current cost of NGS technology is still prohibitive [53]. Therefore, MS/MS will remain a major screening platform; however, it must evolve, as the example of MPS-II shows. As mentioned above, in contrast to several other lysosomal enzymes, an iduronate-2-sulfatase enzyme assay cannot simply be added to the FIA-MS/MS method currently used by most programs already screening for Pompe disease and MPS I. That means that more effort, more equipment and a dedicated DBS punch are required to screen for MPS-II using either LC-MS/MS or fluorometry. Laboratories currently using FIA-MS/MS to screen for LSDs and those planning to screen for LSDs in the future may want to switch to or implement LC-MS/MS as a more forward-looking approach to adding MPS-II. Most NBS programs already screening for X-ALD make use of LC-MS/MS, and their experience further supports the realization coming from many clinical laboratories that LC-MS/MS has become a robust and efficient technology [54] that also allows for high-throughput at a comparable cost in terms of acquisition and maintenance. The possibility of adding more markers for more conditions without the need for more equipment and space makes LC-MS/MS a versatile and cost-effective option for NBS programs that typically must operate within tight budgets.

The assays mentioned in this review primarily fall under the regulatory category of “Laboratory Developed Tests” (LDT), which means that implementation requires NBS laboratories to conduct more comprehensive validation and verification compared to the use of test kits approved by the Federal Drug Administration (FDA) [55]. While FDA-approved tests are generally not superior to LDTs [56], some public health laboratories in the US are prohibited to or do not have the personnel to implement LDTs, which are constraints that continue to significantly delay the consistency of NBS between states, resulting in the further inequitable access of patients to the benefits of early detection and treatment of more conditions.

## 9. Conclusions

Considerable user experience demonstrates no significant incremental maintenance for the additional analytical advantages of LC-MS/MS over FIA-MS/MS applications. LC-MS/MS provides a robust, cost-effective way of measuring the biochemical markers for many disorders which may be added to NBS panels as more therapies become available. It can be an efficient way of measuring enzyme products, particularly those associated with LSDs, with the advantage of the potential for multiplexing screening for several disorders in one test. It is probable that the technology will be increasingly used, even as genomic screening is considered more broadly.

## Figures and Tables

**Table 1:** Comparison of LC-MS/MS to FIA-MS/MS.

Feature	FIA-MS/MS	LC-MS/MS
Equipment	Nitrogen source, Autosampler, one pump with controller, MS/MS, Computer	Nitrogen source, Autosampler, two pumps with binary solvent controller, MS/MS, Computer
Principle	Sample is pumped through tubing from an autosampler to MS/MS via a single pump	Same as that of FIA-MS/MS, except two pumps are used, and an LC column is spliced into the tubing between the autosampler and MS/MS
Cost		Addition of LC to an MS/MS system increases the price of the equipment by ~25%
Chromatography Column	Not required	Required; increases the cost of an MS/MS assay by <1%
Non-maintenance additional hands-on labor		Software-based, automated integration of analyte peaks needs to be double-checked; requires ~10 min per 96–well plate. Replace LC column every 10,000–20,000 samples; requires ~1 h of labor.
Time per DBS analysis	~1.5 min	~2.1 min
Reagents and infrastructure		Solvent use, waste disposal and heat dissipation are similar; space needs may be higher by up to 20%

**Table 2:** LC-MS/MS assays currently used in NBS laboratories or pilot studies associated with the authors^.1^.

Disorder(s)	Marker(s)	Method	First or Second- Tier Test	Comments
MPS, all types [11,12,13]	glycosaminoglycans	LC-MS/MS	second	All NBS labs contract with another lab, except in Italy [18]
MPS-I	α-iduronidase activity	FIA-MS/MS or LC-MS/MS	first	NBS labs in the USA, Taiwan, the Netherlands and regions of Italy use FIA-MS/MS, except Illinois [19] and Utah, which use LC-MS/MS
MPS-II	iduronate-2-sulfatase activity	LC-MS/MS	first	Illinois [20], Taiwan, ScreenPlus (pilot)
MPS-IIIA	heparan N-sulfatase activity	LC-MS/MS	first	ScreenPlus (pilot)
MPS-IIIB	α-N-acetyl- glucosaminidase activity	LC-MS/MS	first	2/3 of Taiwan, ScreenPlus (pilot)
MPS-IVA	galactosamine-6-sulfatase activity	LC-MS/MS	first	Taiwan, ScreenPlus (pilot)
MPS-IVB/ GM1-gangliosidosis	β-galactosidase activity	LC-MS/MS	first	ScreenPlus (pilot)
MPS-VI	arylsulfatase B activity	LC-MS/MS	first	Taiwan, ScreenPlus (pilot)
MPS-VII	β-glucuronidase activity	LC-MS/MS	first	ScreenPlus (pilot)
X-ALD [21,22]	C26-lysophosphatidylcholine	LC-MS/MS or FIA-MS/MS	first- and second-tier	The Netherlands, Taiwan. Connecticut, Illinois, Minnesota, Missouri, North Carolina (pilot), Utah, Washington and 2/3 of Taiwan use first-tier LC-MS/MS; all other US labs use second-tier LC-MS/MS
Krabbe disease	galactosylcerebrosidase activity	FIA-MS/MS or LC-MS/MS	first	Georgia (Pilot), Illinois, Indiana, Kentucky, New York, Ohio, Pennsylvania and Tennessee all use FIA-MS/MS, except Illinois uses LC-MS/MS
Krabbe disease [15,23]	Psychosine	LC-MS/MS	second	Most but not all NBS labs obtain second-tier tests through a contract with another laboratory
Pompe disease	acid α-glucosidase activity	FIA-MS/MS or LC-MS/MS	first	~50% of NBS labs in the USA, 2/3 of Taiwan and regions of Italy all use FIA-MS/MS, except Illinois and 1/3 of Taiwan use LC-MS/MS
Fabry disease	α-galactosidase A activity	FIA-MS/MS or LC-MS/MS	first	Tennessee, New Jersey, Pennsylvania, regions of Italy and 2/3 of Taiwan use FIA-MS/MS; Illinois and 1/3 of Taiwan use LC-MS/MS
Fabry disease	globotriaosyl- sphingosine	LC-MS/MS	second	Used but not relied upon in Italy because it is only abnormal in classic Fabry disease [18]; ScreenPlus (pilot)
Niemann-Pick A/B	acid sphingomyelinase activity	LC-MS/MS	first	Illinois, regions of Italy [18], ScreenPlus (pilot)
Niemann-Pick A/B	lysosphingomyelin, N-palmitoyl-O-phosphocholine-serine (lyso-SM-509)	LC-MS/MS	second	ScreenPlus (pilot); primarily available through a contract with another laboratory
Gaucher	β-Glucocerebrosidase activity	FIA-MS/MS or LC-MS/MS	first	Illinois, ScreenPlus (pilot) and 1/3 of Taiwan use LC-MS/MS; New Jersey, Pennsylvania, Tennessee, regions of Italy and 2/3 of Taiwan use FIA-MS/MS
Gaucher	glucosylsphingosine	LC-MS/MS	second	Italy [18], ScreenPlus (pilot); primarily available through a contract with another laboratory
Congenital adrenal hyperplasia [9,10,24]	17-hydroxy- progesterone, androstenedione, 11-deoxycortisol, 21-deoxycortisol, cortisol	LC-MS/MS	second	Primarily available through a contract with another laboratory
Maple syrup urine disease [25]	allo-isoleucine, isoleucine, leucine, valine, hydroxyproline	LC-MS/MS	second	Primarily available through a contract with another laboratory
Propionic acidemia/methylmalonic acidemias/homocystinuria/ remethylation disorders [16,26,27]	methylmalonic acid, methylcitric acid, total homocysteine, 3-hydroxypropionic acid	LC-MS/MS	second	Primarily available through a contract with another laboratory
SCAD/GA I/GA II/EE [28]	ethylmalonic acid, glutaric acid, 3-hydroxy glutaric acid, 2-hydroxyglutaric acid	LC-MS/MS	second	Primarily available through a contract with another laboratory
Tyrosinemia type I [29]	Succinylacetone	LC-MS/MS	second	Now included in the primary screening test of amino acids and acylcarnitines by FIA-MS/MS
Neuronal ceroid lipofuscinosis 2 [30]	tripeptidyl protease 1 activity	LC-MS/MS	first	2/3 of Taiwan, ScreenPlus (pilot)
Wolman disease, cholesterol ester storage disease	lysosomal acid lipase activity	LC-MS/MS	first	ScreenPlus (pilot)
Niemann-Pick C	bile acid B	LC-MS/MS	first	ScreenPlus (pilot)
α-Mannosidosis	α-mannosidosis activity	LC-MS/MS	first	ScreenPlus (pilot)
MLD	C16:0-sulfatide	LC-MS/MS	first	ArchimedLife, ScreenPlus (pilot)
MLD [31]	arylsulfatase A activity	LC-MS/MS	second	ScreenPlus (pilot)
CTX	cholestanetetrol glucuronide	LC-MS/MS or FIA-MS/MS	first	ScreenPlus (pilot) uses LC-MS/MS; Amsterdam UMC (pilot) compares LC-MS/MS vs. FIA-MS/MS
CTX	7-α-hydroxy-4-cholesten-3-one; 7-α,12 α-dihydroxycholest-4-en-3-one	LC-MS/MS	second	Primarily available through a contract with another laboratory

^1^ Abbreviations: CTX, cerebrotendinous xanthomatosis; EE, ethylmalonic encephalopathy; GA I, glutaric acidemia type I; GA II, glutaric acidemia type II; MLD, metachromatic leukodystrophy; MPS, mucopolysaccharidosis; SCAD, short-chain acyl-CoA dehydrogenase deficiency; X-ALD, X-linked adrenoleukodystrophy.

**Table 3:** LC-MS/MS equipment maintenance. Provided information reflects the authors’ experience in their laboratories.

NBS Laboratory	LC-MS/MS Assay	Years in Use	Sample Injections before Column Replacement	Preventive Maintenance per Year	Additional Servicing per Year
Amsterdam UMC, The Netherlands	CTX	1.5	10 k (guard); 10–20 k (main)	2 (MS/MS); 1 (LC)	none
Connecticut DPH	X-ALD	7	11 k (guard); 22 k (main)	2 (MS/MS); 1 (LC)	none
Illinois DPH	MPS-I, MPS-II, Gaucher, Fabry, Krabbe, NP-A/B, Pompe	9 (5 for MPS-II, Krabbe)	3–5.5 k (guard); 11–13 k (main)	2 (MS/MS); 1 (LC)	UPLC pump seal replaced once (3–4 hr down time)
Illinois DPH	X-ALD	3	2–3 k (guard); 20–22 k (main)	2 (MS/MS); 1 (LC)	none
Minnesota DPH	X-ALD	5	7 k (guard); 13 k (main)	2–3 (MS/MS); 1 (LC)	parts replacement similar to FIA-MS/MS
Missouri DPH	X-ALD	0.5	8 k (guard); 20 k (main)	2 (MS/MS); 1 (LC)	none
National Taiwan University Hospital	MPS-II, MPS-IIIB, MPS-IVA, MPS-VI, X-ALD, CLN2	4 (MPS II, IIIB, IVA, VI, CLN2) and 6 yr (ALD)	5 k (guard); 10–20 k (main)	2	none
Chinese Foundation of Health, Taiwan	MPS-II, MPS-IVA, MPS-VI, X-ALD	4 (ALD) and 6 yr (MPS II, IVA, VI)	10 k (guard); 10–20 k (main)	2–4	only for autosampler
Taipei Institute of Health, Taiwan	MPS-II, MPS-IIIB, MPS-IVA, MPS-VI, X-ALD, CLN2,	4 (MPS-II, X-ALD) 2 (MPS-IVA, MPS-VI, CLN2)	8 k (guard); 40–50 k (main)	2	only for autosampler
University of Washington	MPS-II MPS-IIIB, MPS-IVA, MPS-VI, MPS-VII	2.5	3.3 k (guard); 10 k (main)	2	none
University of Washington	MLD, CTX	1.5	3.3 k (guard); 10 k (main)	1	none
University Hospital of Padova, Italy	Fabry, Gaucher, Pompe, MPS-I, NP-A/B, Krabbe	1	3–5 k (guard); 13 k (main)	2	UPLC and autosampler repaired once (1–2 days downtime)
Utah DOH	X-ALD	1.5	14 k (main)	2 (MS/MS); 1 (LC)	none

Abbreviations: CLN2, neuronal ceroid lipofuscinosis 2; CTX, cerebrotendinous xanthomatosis; DPH, department of public health; k, 1000; MPS, mucopolysaccharidosis; NP-A/B, Niemann-Pick disease A or B; UPLC, ultra-performance liquid chromatography; X-ALD, X-linked adreno-leukodystrophy.

## Data Availability

Not applicable.

## References

[1] Chace D.H. (2009). Mass spectrometry in newborn and metabolic screening: Historical perspective and future directions. J. Mass Spectrom..

[2] Gelb M.H. (2018). Newborn Screening for Lysosomal Storage Diseases: Methodologies, Screen Positive Rates, Normalization of Datasets, Second-Tier Tests, and Post-Analysis Tools. Int. J. Neonatal Screen..

[3] La Marca G., Casetta B., Malvagia S., Guerrini R., Zammarchi E. (2009). New strategy for the screening of lysosomal storage disorders: The use of the online trapping-and-cleanup liquid chromatography/mass spectrometry. Anal. Chem..

[4] Metz T.F., Mechtler T.P., Orsini J.J., Martin M., Shushan B., Herman J.L., Ratschmann R., Item C.B., Streubel B., Herkner K.R. (2011). Simplified newborn screening protocol for lysosomal storage disorders. Clin. Chem..

[5] Scott C.R., Elliott S., Hong X., Huang J.Y., Kumar A.B., Yi F., Pendem N., Chennamaneni N.K., Gelb M.H. (2020). Newborn Screening for Mucopolysaccharidoses: Results of a Pilot Study with 100,000 Dried Blood Spots. J. Pediatr..

[6] Hong X., Daiker J., Sadilek M., Ruiz-Schultz N., Kumar A.B., Norcross S., Dansithong W., Suhr T., Escolar M.L., Ronald Scott C. (2021). Toward newborn screening of metachromatic leukodystrophy: Results from analysis of over 27,000 newborn dried blood spots. Genet. Med..

[7] Jiang X., Sidhu R., Orsini J.J., Farhat N.Y., Porter F.D., Berry-Kravis E., Schaffer J.E., Ory D.S. (2019). Diagnosis of niemann-pick C1 by measurement of bile acid biomarkers in archived newborn dried blood spots. Mol. Genet. Metab..

[8] Matern D., Tortorelli S., Oglesbee D., Gavrilov D., Rinaldo P. (2007). Reduction of the false-positive rate in newborn screening by implementation of MS/MS-based second-tier tests: The Mayo Clinic experience (2004–2007). J. Inherit. Metab. Dis..

[9] Lacey J.M., Minutti C.Z., Magera M.J., Tauscher A.L., Casetta B., McCann M., Lymp J., Hahn S.H., Rinaldo P., Matern D. (2004). Improved specificity of newborn screening for congenital adrenal hyperplasia by second-tier steroid profiling using tandem mass spectrometry. Clin. Chem..

[10] Minutti C.Z., Lacey J.M., Magera M.J., Hahn S.H., McCann M., Schulze A., Cheillan D., Dorche C., Chace D.H., Lymp J.F. (2004). Steroid profiling by tandem mass spectrometry improves the positive predictive value of newborn screening for congenital adrenal hyperplasia. J. Clin. Endocrinol. Metab..

[11] Herbst Z.M., Urdaneta L., Klein T., Fuller M., Gelb M.H. (2020). Evaluation of Multiple Methods for Quantification of Glycosaminoglycan Biomarkers in Newborn Dried Blood Spots from Patients with Severe and Attenuated Mucopolysaccharidosis-I. Int. J. Neonatal Screen..

[12] Peck D.S., Lacey J.M., White A.L., Pino G., Studinski A.L., Fisher R., Ahmad A., Spencer L., Viall S., Shallow N. (2020). Incorporation of Second-Tier Biomarker Testing Improves the Specificity of Newborn Screening for Mucopolysaccharidosis Type I. Int. J. Neonatal Screen..

[13] Herbst Z.M., Urdaneta L., Klein T., Burton B.K., Basheeruddin K., Liao H.C., Fuller M., Gelb M.H. (2022). Evaluation of Two Methods for Quantification of Glycosaminoglycan Biomarkers in Newborn Dried Blood Spots from Patients with Severe and Attenuated Mucopolysaccharidosis Type II. Int. J. Neonatal Screen..

[14] ACHDNC 12–13 May 2022 Meeting. https://www.hrsa.gov/advisory-committees/heritable-disorders/meetings/may-12-2022.

[15] Guenzel A.J., Turgeon C.T., Nickander K.K., White A.L., Peck D.S., Pino G.B., Studinski A.L., Prasad V.K., Kurtzberg J., Escolar M.L. (2020). The critical role of psychosine in screening, diagnosis, and monitoring of Krabbe disease. Genet. Med..

[16] Gavrilov D.K., Piazza A.L., Pino G., Turgeon C., Matern D., Oglesbee D., Raymond K., Tortorelli S., Rinaldo P. (2020). The Combined Impact of CLIR Post-Analytical Tools and Second Tier Testing on the Performance of Newborn Screening for Disorders of Propionate, Methionine, and Cobalamin Metabolism. Int. J. Neonatal Screen..

[17] Minter Baerg M.M., Stoway S.D., Hart J., Mott L., Peck D.S., Nett S.L., Eckerman J.S., Lacey J.M., Turgeon C.T., Gavrilov D. (2018). Precision newborn screening for lysosomal disorders. Genet. Med..

[18] Burlina A.B., Polo G., Rubert L., Gueraldi D., Cazzorla C., Duro G., Salviati L., Burlina A.P. (2019). Implementation of Second-Tier Tests in Newborn Screening for Lysosomal Disorders in North Eastern Italy. Int. J. Neonatal Screen..

[19] Burton B.K., Charrow J., Hoganson G.E., Waggoner D., Tinkle B., Braddock S.R., Schneider M., Grange D.K., Nash C., Shryock H. (2017). Newborn Screening for Lysosomal Storage Disorders in Illinois: The Initial 15-Month Experience. J. Pediatr..

[20] Burton B.K., Hickey R., Hitchins L. (2020). Newborn Screening for Mucopolysaccharidosis Type II in Illinois: An Update. Int. J. Neonatal Screen..

[21] Moser A.B., Jones R.O., Hubbard W.C., Tortorelli S., Orsini J.J., Caggana M., Vogel B.H., Raymond G.V. (2016). Newborn Screening for X-Linked Adrenoleukodystrophy. Int. J. Neonatal Screen..

[22] Tortorelli S., Turgeon C.T., Gavrilov D.K., Oglesbee D., Raymond K.M., Rinaldo P., Matern D. (2016). Simultaneous Testing for 6 Lysosomal Storage Disorders and X-Adrenoleukodystrophy in Dried Blood Spots by Tandem Mass Spectrometry. Clin. Chem..

[23] Herbst Z., Turgeon C.T., Biski C., Khaledi H., Shoemaker N.B., DeArmond P.D., Smith S., Orsini J., Matern D., Gelb M.H. (2020). Achieving Congruence among Reference Laboratories for Absolute Abundance Measurement of Analytes for Rare Diseases: Psychosine for Diagnosis and Prognosis of Krabbe Disease. Int. J. Neonatal Screen..

[24] Janzen N., Sander S., Terhardt M., Steuerwald U., Peter M., Das A.M., Sander J. (2011). Rapid steroid hormone quantification for congenital adrenal hyperplasia (CAH) in dried blood spots using UPLC liquid chromatography-tandem mass spectrometry. Steroids.

[25] Oglesbee D., Sanders K.A., Lacey J.M., Magera M.J., Casetta B., Strauss K.A., Tortorelli S., Rinaldo P., Matern D. (2008). Second-tier test for quantification of alloisoleucine and branched-chain amino acids in dried blood spots to improve newborn screening for maple syrup urine disease (MSUD). Clin. Chem..

[26] La Marca G., Malvagia S., Pasquini E., Innocenti M., Donati M.A., Zammarchi E. (2007). Rapid 2nd-tier test for measurement of 3-OH-propionic and methylmalonic acids on dried blood spots: Reducing the false-positive rate for propionylcarnitine during expanded newborn screening by liquid chromatography-tandem mass spectrometry. Clin. Chem..

[27] Turgeon C.T., Magera M.J., Cuthbert C.D., Loken P.R., Gavrilov D.K., Tortorelli S., Raymond K.M., Oglesbee D., Rinaldo P., Matern D. (2010). Determination of total homocysteine, methylmalonic acid, and 2-methylcitric acid in dried blood spots by tandem mass spectrometry. Clin. Chem..

[28] Adhikari A.N., Currier R.J., Tang H., Turgeon C.T., Nussbaum R.L., Srinivasan R., Sunderam U., Kwok P.Y., Brenner S.E., Gavrilov D. (2020). Genomic Analysis of Historical Cases with Positive Newborn Screens for Short-Chain Acyl-CoA Dehydrogenase Deficiency Shows That a Validated Second-Tier Biochemical Test Can Replace Future Sequencing. Int. J. Neonatal Screen..

[29] Magera M.J., Gunawardena N.D., Hahn S.H., Tortorelli S., Mitchell G.A., Goodman S.I., Rinaldo P., Matern D. (2006). Quantitative determination of succinylacetone in dried blood spots for newborn screening of tyrosinemia type I. Mol. Genet. Metab..

[30] Khaledi H., Liu Y., Masi S., Gelb M.H. (2018). Detection of Infantile Batten Disease by Tandem Mass Spectrometry Assay of PPT1 Enzyme Activity in Dried Blood Spots. Anal. Chem..

[31] Hong X., Kumar A.B., Daiker J., Yi F., Sadilek M., De Mattia F., Fumagalli F., Calbi V., Damiano R., Della Bona M. (2020). Leukocyte and Dried Blood Spot Arylsulfatase A Assay by Tandem Mass Spectrometry. Anal. Chem..

[32] Burton B.K., Charrow J., Hoganson G.E., Fleischer J., Grange D.K., Braddock S.R., Hitchins L., Hickey R., Christensen K.M., Groepper D. (2020). Newborn Screening for Pompe Disease in Illinois: Experience with 684,290 Infants. Int. J. Neonatal Screen..

[33] Chien Y.H., Lee N.C., Chen P.W., Yeh H.Y., Gelb M.H., Chiu P.C., Chu S.Y., Lee C.H., Lee A.R., Hwu W.L. (2020). Newborn screening for Morquio disease and other lysosomal storage diseases: Results from the 8-plex assay for 70,000 newborns. Orphanet Rare Dis..

[34] Bilyeu H., Washburn J., Vermette L., Klug T. (2020). Validation and Implementation of a Highly Sensitive and Efficient Newborn Screening Assay for Mucopolysaccharidosis Type II. Int. J. Neonatal Screen..

[35] Haynes C.A., De Jesus V.R. (2016). Simultaneous quantitation of hexacosanoyl lysophosphatidylcholine, amino acids, acylcarnitines, and succinylacetone during FIA-ESI-MS/MS analysis of dried blood spot extracts for newborn screening. Clin. Biochem..

[36] Hong X., Sadilek M., Gelb M.H. (2020). A highly multiplexed biochemical assay for analytes in dried blood spots: Application to newborn screening and diagnosis of lysosomal storage disorders and other inborn errors of metabolism. Genet. Med..

[37] Tan M.A., Dean C.J., Hopwood J.J., Meikle P.J. (2008). Diagnosis of metachromatic leukodystrophy by immune quantification of arylsulphatase A protein and activity in dried blood spots. Clin. Chem..

[38] Biffi A., Montini E., Lorioli L., Cesani M., Fumagalli F., Plati T., Baldoli C., Martino S., Calabria A., Canale S. (2013). Lentiviral Hematopoietic Stem Cell Gene Therapy Benefits Metachromatic Leukodystrophy. Science.

[39] Vaz F.M., Bootsma A.H., Kulik W., Verrips A., Wevers R.A., Schielen P.C., DeBarber A.E., Huidekoper H.H. (2017). A newborn screening method for cerebrotendinous xanthomatosis using bile alcohol glucuronides and metabolite ratios. J. Lipid Res..

[40] Hong X., Daiker J., Sadilek M., DeBarber A.E., Chiang J., Duan J., Bootsma A.H., Huidekoper H.H., Vaz F.M., Gelb M.H. (2020). Toward Newborn Screening of Cerebrotendinous Xanthomatosis: Results of a Biomarker Research Study Using 32,000 Newborn Dried Blood Spots. Genet. Med. Off. J. Am. Coll. Med. Genet..

[41] Yi F., Hong X., Kumar A.B., Zong C., Boons G.J., Scott C.R., Turecek F., Robinson B.H., Gelb M.H. (2018). Detection of mucopolysaccharidosis III-A (Sanfilippo Syndrome-A) in dried blood spots (DBS) by tandem mass spectrometry. Mol. Genet. Metab..

[42] Meikle P.J., Grasby D.J., Dean C.J., Lang D.L., Bockmann M., Whittle A.M., Fietz M.J., Simonsen H., Fuller M., Brooks D.A. (2006). Newborn screening for lysosomal storage disorders. Mol. Genet. Metab..

[43] Singh R., Chopra S., Graham C., Langer M., Ng R., Ullal A.J., Pamula V.K. (2020). Emerging Approaches for Fluorescence-Based Newborn Screening of Mucopolysaccharidoses. Diagnostics.

[44] Collins C.J., Chang I.J., Jung S., Dayuha R., Whiteaker J.R., Segundo G.R.S., Torgerson T.R., Ochs H.D., Paulovich A.G., Hahn S.H. (2018). Rapid Multiplexed Proteomic Screening for Primary Immunodeficiency Disorders from Dried Blood Spots. Front. Immunol..

[45] Collins C.J., Yi F., Dayuha R., Duong P., Horslen S., Camarata M., Coskun A.K., Houwen R.H.J., Pop T.L., Zoller H. (2021). Direct Measurement of ATP7B Peptides Is Highly Effective in the Diagnosis of Wilson Disease. Gastroenterology.

[46] Kroll C.A., Ferber M.J., Dawson B.D., Jacobson R.M., Mensink K.A., Lorey F., Sherwin J., Cunningham G., Rinaldo P., Matern D. (2006). Retrospective determination of ceruloplasmin in newborn screening blood spots of patients with Wilson disease. Mol. Genet. Metab..

[47] Wasserstein M.P. ScreenPlus: A Comprehensive, Flexible, Multi-Disorder Newborn Screening Program. https://einsteinmed.edu/research/screenplus.

[48] Sanders K.A., Gavrilov D.K., Oglesbee D., Raymond K.M., Tortorelli S., Hopwood J.J., Lorey F., Majumdar R., Kroll C.A., McDonald A.M. (2020). A Comparative Effectiveness Study of Newborn Screening Methods for Four Lysosomal Storage Disorders. Int. J. Neonatal Screen..

[49] Bailey D.B., Porter K.A., Andrews S.M., Raspa M., Gwaltney A.Y., Peay H.L. (2021). Expert Evaluation of Strategies to Modernize Newborn Screening in the United States. JAMA Netw. Open.

[50] Orsini J.J., Kay D.M., Saavedra-Matiz C.A., Wenger D.A., Duffner P.K., Erbe R.W., Biski C., Martin M., Krein L.M., Nichols M. (2016). Newborn screening for Krabbe disease in New York State: The first eight years’ experience. Genet. Med..

[51] ClinVar GALC [Gene]. https://www.ncbi.nlm.nih.gov/clinvar/?gr=0&term=GALC%5Bgene%5D&redir=gene.

[52] Muriello M., Basel D. (2022). Rapid Exome and Genome Sequencing in the Intensive Care Unit. Crit. Care Clin..

[53] Kingsmore S.F., Smith L.D., Kunard C.M., Bainbridge M., Batalov S., Benson W., Blincow E., Caylor S., Chambers C., Del Angel G. (2022). A genome sequencing system for universal newborn screening, diagnosis, and precision medicine for severe genetic diseases. Am. J. Hum. Genet..

[54] Seger C., Salzmann L. (2020). After another decade: LC-MS/MS became routine in clinical diagnostics. Clin. Biochem..

[55] Graden K.C., Bennett S.A., Delaney S.R., Gill H.E., Willrich M.A.V. (2021). A High-Level Overview of the Regulations Surrounding a Clinical Laboratory and Upcoming Regulatory Challenges for Laboratory Developed Tests. Lab. Med..

[56] Kim A.S., Bartley A.N., Bridge J.A., Kamel-Reid S., Lazar A.J., Lindeman N.I., Long T.A., Merker J.D., Rai A.J., Rimm D.L. (2018). Comparison of Laboratory-Developed Tests and FDA-Approved Assays for BRAF, EGFR, and KRAS Testing. JAMA Oncol..

